# The Endoplasmic Reticulum Is a Key Battleground between Phytoplasma Aggression and Host Plant Defense

**DOI:** 10.3390/cells12162110

**Published:** 2023-08-21

**Authors:** Junichi Inaba, Bo Min Kim, Yan Zhao, Andrew M. Jansen, Wei Wei

**Affiliations:** 1 Molecular Plant Pathology Laboratory, Beltsville Agricultural Research Center, Agricultural Research Service, United States Department of Agriculture, Beltsville, MD 20705, USA; junichi.inaba@usda.gov (J.I.); bomin.kim@usda.gov (B.M.K.); yan.zhao@usda.gov (Y.Z.); 2 Electron and Confocal Microscopy Unit, Beltsville Agricultural Research Center, Agricultural Research Service, United States Department of Agriculture, Beltsville, MD 20705, USA; andrew.jansen@usda.gov

**Keywords:** endoplasmic reticulum (ER), ER stress, unfolded protein response (UPR), phytoplasma, ER-resident proteins

## Abstract

Phytoplasmas are intracellular plant pathogens that heavily rely on host cell nutrients for survival and propagation due to their limited ability to synthesize essential substrates. The endoplasmic reticulum (ER), which plays a vital role in various cellular processes, including lipid and protein biosynthesis, is an attractive target for numerous intracellular pathogens to exploit. This study investigated the impact of potato purple top (PPT) phytoplasma infection on the ER in tomato plants. Abnormal accumulation of ER-resident proteins, disrupted ER network structures, and formation of protein aggregates in the phloem were observed using confocal microscopy and transmission electron microscopy, indicating a phytoplasma-infection-induced disturbance in ER homeostasis. The colocalization of phytoplasmas with the accumulated ER-resident proteins suggests an association between ER stress, unfolded protein response (UPR) induction, and phytoplasma infection and colonization, with the ER stress response likely contributing to the host plant’s defense mechanisms. Quantitative real-time PCR revealed a negative correlation between ER stress/UPR activation and PPT phytoplasma titer, implying the involvement of UPR in curbing phytoplasma proliferation. Inducing ER stress and activating the UPR pathway effectively decreased phytoplasma titer, while suppressing the ER-resident protein, binding immunoglobulin protein (BiP) increased phytoplasma titer. These results highlight the ER as an intracellular battleground where phytoplasmas exploit host components for survival and multiplication, while host plants deploy defense mechanisms to counteract the invasion. Understanding the intricate interactions between phytoplasmas and plant hosts at the subcellular level, particularly within the ER, provides valuable insights for developing new strategies to control phytoplasma diseases.

## 1. Introduction

Phytoplasmas are a unique group of intracellular bacteria that infect plants and cause numerous diseases. They belong to the class Mollicutes and are closely related to mycoplasmas, which are known for causing diseases in humans and animals. Unlike most bacteria, phytoplasmas lack a cell wall, which gives them a pleomorphic shape [1,2]. As intracellular bacteria, phytoplasmas reside and multiply within the plant phloem, a specialized vascular tissue comprising cell types such as sieve elements, companion cells, and phloem parenchyma cells, which facilitate nutrient transport, primarily sugars and other organic molecules, throughout the plant. Phytoplasma infection induces various symptoms, such as stunted growth, witches’-broom, leaf curling, and chlorosis [3,4]. These symptoms can significantly reduce crop yield and quality, resulting in considerable economic impact on agriculture, horticulture, and forestry. In nature, phytoplasmas are transmitted from one plant to another by phloem sap-feeding insect vectors, including leafhoppers, planthoppers, and psyllids [5].

Phytoplasmas have evolved various strategies to survive and thrive within host plant cells. Like other intracellular pathogens, phytoplasmas possess smaller genomes compared to free-living bacteria. Ample available phytoplasma genome sequences demonstrated that phytoplasmas lack certain genes required for independent survival, rendering them reliant on their host for specific metabolic processes and nutrients. It is believed that phytoplasmas exploit the plant’s phloem system to multiply and spread throughout the plant and cause disease [6,7]. However, how phytoplasmas adapt to the intracellular environment of the phloem sieve elements and establish persistent infections remains unknown, as studies of these bacteria have been hindered due to their unculturable nature.

The endoplasmic reticulum (ER) is a crucial organelle that plays a significant role in various cellular processes, including protein synthesis, modification, folding, protein quality control, and lipid metabolism. The ER membrane is also the site of numerous enzymes and proteins involved in these processes [8,9]. One of the most important classes of ER-resident proteins is the molecular chaperones, which help to fold newly synthesized proteins and maintain the proper conformation of proteins in the ER. These chaperones include the ER luminal binding immunoglobulin protein (BiP) and the protein disulfide isomerase (PDI) [10,11]. Another essential class of ER-resident proteins is the ER-associated degradation (ERAD) machinery that is responsible for recognizing and degrading misfolded or unassembled proteins in the ER. The ERAD machinery includes the ER-resident E3 ubiquitin ligases, which target misfolded proteins for ubiquitination and subsequent degradation by the proteasome [12].

The ER consists of an interconnected network of membrane-bound tubules and flattened sacs (cisternae), extending throughout the cytoplasm and continuous with the nuclear envelope [8,13]. Many plant pathogens, including bacteria, viruses, and fungi, can hijack the ER for their own replication and survival. For example, bacterial pathogens such as *Pseudomonas syringae* and *Xanthomonas campestris* can secrete effector proteins that target the ER and manipulate its functions to facilitate bacterial infection and colonization [14,15]. Similarly, plant viruses such as tobacco mosaic virus (TMV) and potato virus X (PVX) can co-opt the ER membrane to create replication compartments and utilize ER-resident proteins for their own replication [16,17]. The interaction between plant pathogens and the ER is complex and dynamic, with pathogens utilizing a variety of mechanisms to exploit this organelle for their own benefit [18].

Recent studies have suggested that phytoplasma infection also alters the ER’s morphology and induces the unfolded protein response (UPR), a cellular mechanism that maintains ER homeostasis [19,20,21]. Building upon these findings, the present study aimed to investigate the impact of potato purple top (PPT) phytoplasma infection on the ER and its resident proteins and UPR in tomato plants. To achieve this, an antibody was raised against an immunodominant membrane protein (IDP) of the PPT phytoplasma, facilitating clear visualization of phytoplasma presence. IDP occupies a significant portion of cellular membrane across various phytoplasma species, and its interactions with host plants and insect vectors have been extensively studied [22,23]. Moreover, for precise visualization of the ER, a well-established and universally recognized marker, the HDEL signal, was employed. HDEL, denoting the peptide sequence His–Asp–Glu–Leu, functions both as a retention and retrieval signal for proteins destined for the ER lumen in eukaryotic cells. This distinctive signal not only indicates a state of ER homeostasis but also offers the capability to effectively visualize the intricate network structure of the ER [24,25].

Our findings, through confocal microscopy along with anti-PPT-IDP and anti-HDEL antibodies, unveiled that there was an excessive accumulation of ER-resident proteins in sieve elements of phytoplasma-infected plants. Such a phenomenon indicated phytoplasma infection induced ER stress, which in turn triggered UPR activation. The spatial colocalization of phytoplasmas with ER-resident proteins prompted the hypothesis that ER and its resident proteins might be associated with phytoplasma multiplication and survival. The hypothesis was supported by two lines of evidence: the activation of UPR and upregulation of ER-resident proteins including BiP via exogenous application of UPR chemical inducers led to the inhibition of phytoplasma multiplication; and, conversely, the knockdown of BiP led to an increase in phytoplasma titer. Additionally, the present study revealed a positive interaction between UPR and the salicylic acid (SA)-dependent defense pathway in the host plant’s response to phytoplasma infection. The findings and the hypothesis derived from this study provide clues to the eventual elucidation of the mechanisms of how phytoplasmas interact with the host and utilize host components, such as ER and its resident proteins, for their colonization and proliferation. This knowledge will in turn serve as a basis for developing novel strategies for disease management through targeting the UPR pathway to enhance plant resistance against phytoplasmas and other pathogens.

## 2. Materials and Methods

### 2.1. Phytoplasma Strains and Graft Inoculation

The Columbia Basin PPT phytoplasma (also known as BLTVA) was maintained in tomato plants under greenhouse conditions, with 16 h of light and 8 h of darkness. To investigate the effects of the PPT phytoplasma on tomato plants, the tomato cultivar Money Maker was used, and the phytoplasma infection was established through grafting inoculation, following previously established protocols [26]. Recipient plants, representing healthy tomato seedlings at the four-leaf stage, were utilized as rootstocks. For the phytoplasma inocula (scions), infected shoots displaying witches’-broom symptoms were employed. To prepare the inoculum, a freshly cut shoot was trimmed into a wedge shape at its lower end, which was then inserted into a cleft cut in the main stem of the recipient plant. Firm fixation of the graft union was ensured using a plastic clip. Subsequently, the grafted plants were placed on a mist bench for a week, followed by transfer to a climate-controlled greenhouse (maintaining conditions of 25 °C and 70–80% humidity). The day of graft inoculation was designated as day 0 as this is the time point when the host plant was exposed to the phytoplasma inoculum.

### 2.2. DNA and RNA Extraction

To detect phytoplasma, total DNA was extracted from leaf tissues by using a modified CTAB method [27]. The procedure included grinding 0.1 g of tomato leaves with liquid nitrogen, followed by mixing the samples with 1 mL of 100 mM Tris pH 8.0, 1.4 M NaCl, 50 mM EDTA, 2.5% CTAB and 1% polyvinylpyrrolidone (PVP-40). The mixture was incubated at 65 °C for 30 min, and after chloroform and isopropanol precipitations, the DNA pellets were resuspended in 400 µL of water and treated with RNase A. Purified DNAs were obtained through phenol and ethanol precipitations, followed by 70% ethanol washes, and used for further analysis.

For the isolation of plant RNAs, Trizol reagent was employed according to the manufacturer’s protocol (Invitrogen, Waltham, MA, USA); 0.1 g of tomato leaf tissues was ground with liquid nitrogen, and 1 mL of Trizol reagent was added to the samples. After a 10 min incubation at room temperature and centrifugation at 4 °C following the addition of chloroform, the supernatants were transferred and subjected to isopropanol precipitation and 70% ethanol wash. The resulting RNA pellets were dried, resuspended in water, and ready for subsequent experiments.

### 2.3. Production of Polyclonal Antibodies against the Immunodominant Membrane Protein (IDP) of PPT Phytoplasma

The antigen used for producing polyclonal antibodies was a recombinant PPT phytoplasma IDP (PPT-IDP, for the description of IDP, please see Section 1, Introduction). To obtain the full-length gene-encoding PPT-IDP, genomic DNA was extracted from PPT-infected tomato plants using a previously established protocol (see above). PCR amplification was then performed using specific primer pairs PPT-IDP-F and PPT-IDP-R (Supplementary Table S1), designed based on the PPT-IDP gene sequence (Accession number JANHJP000000000) [28]. The pDONR221 entry vector (Invitrogen, Waltham, MA, USA) in the Gateway cloning system was utilized to create a recombinant entry clone by recombining the PPT-IDP PCR product with the vector using BP Clonase II enzyme (Thermo Fisher Scientific, Waltham, MA, USA). The pDEST17 expression vector (Invitrogen, Waltham, MA, USA) containing an N-terminal 6xHis tag was used for protein purification. The PPT-IDP gene was transferred with LR Clonase II enzyme from the entry clone to the pDEST17 vector, resulting in the creation of the pDEST17-PPT-IDP expression construct. The construct was transformed into *E. coli* LOBSTR-BL21(DE3)-RIL competent cells (Kerafast, Boston, MA, USA) and induced with 1 mM IPTG to express the IDP protein, which was then harvested after 16 h. The recombinant PPT-IDP protein was solubilized using detergent and purified using Ni-NTA Agarose (Qiagen, Hilden, Germany), following the manufacturer’s instructions. SDS-PAGE was conducted to confirm the presence and purity of the PPT-IDP protein. The purified protein was sent to Pacific Immunology (Ramona, CA, USA) to produce polyclonal antibodies in rabbits, and the purified IgG obtained from the rabbits was subsequently used for further experiments.

### 2.4. Western Blot Analysis

For the Western blot analysis, the anti-PPT-IDP antibody (synthesized for this study) and anti-BiP antibody (Agrisera, Vännäs, Sweden) were used. Total proteins were extracted from leaves of PPT-phytoplasma-infected and healthy tomato plants, respectively, using a previously described method [29]. The proteins were separated on a Novex™ WedgeWell™ 4 to 20% Tris–Glycine gel (Invitrogen, Waltham, MA, USA). Subsequently, the gel was transferred onto a PVDF membrane using Tris–Glycine Transfer buffer (25 mM Tris, 192 mM glycine, 0.1% SDS) at 400 mA for 90 min. After the transfer, the membrane was blocked with TBS-T (50 mM Tris-HCl, pH 7.5, 150 mM NaCl, 0.1% Tween-20) containing 5% nonfat dry milk. The membrane was then incubated with the anti-PPT-IDP or anti-BiP antibody at 4 °C overnight. Detection of the target proteins was achieved using an anti-rabbit or an anti-goat secondary antibody conjugated with alkaline phosphatase (Promega, Madison, WI, USA, and Thermo Fisher Scientific, Waltham, MA, USA, respectively) and visualized using a colorimetric method.

### 2.5. Visualization and Localization of ER-Resident Proteins and Phytoplasmas in Plants by Immunostaining

For the visualization and localization of ER-resident proteins in infected plants, as well as the identification of phytoplasmas within the tissues, immunostaining was conducted. Paraffin-embedded sections were prepared from the main stems of tomato plants infected with PPT phytoplasma. The stems were fixed with 4% paraformaldehyde, embedded in paraffin, and sectioned to a thickness of 10 μm using a Leitz-1512 Microtome. To visualize the presence of ER-resident proteins and localization of PPT phytoplasmas, deparaffinized sections were immunostained with primary anti-HDEL antibody (Invitrogen, Waltham, MA, USA), anti-BiP antibody (Agrisera, Vännäs, Sweden), and anti-PPT-IDP antibody, respectively. Fluorescently labeled secondary antibodies were used to detect the primary antibodies, including an anti-mouse antibody labeled with Alexa Fluor™ 405 or Alexa Fluor™ 488 (Thermo Fisher Scientific, Waltham, MA, USA), an anti-goat antibody labeled with Alexa Fluor™ 488, and anti-rabbit antibodies labeled with Alexa Fluor™ 488 or Alexa Fluor^®^ 405 (Thermo Fisher Scientific, Waltham, MA, USA). Fluorescent images were captured using a Zeiss LSM710 confocal laser scanning microscopy (CLSM) system (Carl Zeiss AG, Jena, Germany) with an excitation wavelength of 405 nm and 488 nm, and 40–150 z-stack images and maximum intensity projections were obtained using Zeiss Zen 2012 Pro software. Based on the histogram function of Adobe Photoshop software, the fluorescence intensity was assessed (https://helpx.adobe.com/photoshop/using/viewing-histograms-pixel-values.html) (accessed on 20 June 2023).

### 2.6. Transmission Electron Microscopy (TEM)

The stem samples were harvested and processed for sectioning using a resin embedding technique, following established protocols [4]. The specimens underwent postfixation with osmium tetroxide and staining of ultrathin sections with lead citrate and uranyl acetate. TEM images were captured using a Hitachi HT-7700 transmission electron microscope (Hitachi, Tokyo, Japan).

### 2.7. Application of Unfolded Protein Response (UPR) Chemical Inducers to Plants

To investigate the effect of unfolded protein response (UPR) activation on PPT-phytoplasma-infected tomato plants, exogenous chemical treatments were conducted. Young branches of the PPT-phytoplasma-infected plants were incubated in ½ MS medium with dithiothreitol (DTT, 2.5 mM) and Tunicamycin (Tm, 2.5 µg/mL), respectively. After the 24 h incubation period, the leaves were harvested for further gene expression analysis, while the leaves treated for 5 days were used for the measurement of PPT phytoplasma titer.

### 2.8. Assessment of the Expression Levels of Targeted UPR and SA Marker Genes by Real-Time Quantitative Reverse Transcription PCR (qRT-PCR)

From the extracted RNA, cDNAs were synthesized using the AffinityScript Multi-Temperature cDNA synthesis kit (Agilent Technologies, Santa Clara, CA, USA). Real-time qRT-PCR was performed using the AriaMx Real-Time PCR System (Agilent Technologies, Santa Clara, CA, USA), along with Brilliant SYBR Green QPCR Master Mix (Agilent Technologies, Santa Clara, CA, USA). The specific primers for tomato BiP, bZIP60, bZIP17, PR1, PR5, and NPR1 genes used for the qRT-PCR reactions are listed in Supplementary Table S1. In qRT-PCR, the same operating conditions as qPCR for phytoplasma titer measurement were applied (see above). To analyze gene expression fold changes, the expression levels were normalized using tomato tubulin as an internal control. At least three independent RNA samples were included in the analysis to ensure the robustness and reliability of the results. Statistical analysis was performed using the Student’s *t* test in Microsoft Excel (Microsoft Corporation, Seattle, WA, USA) to determine the significance of differences between groups.

### 2.9. Measurement of Phytoplasma Titer by Quantitative PCR (qPCR)

Phytoplasma titer was assessed by qPCR. The host-to-phytoplasma DNA ratio was determined using the AriaMx Real-Time PCR System, which utilized Brilliant SYBR Green QPCR Master Mix (Agilent Technologies, Santa Clara, CA, USA). The 16S rRNA gene fragment from PPT phytoplasma DNA and tomato actin (internal control) fragment from the total DNAs were amplified using MPPLPPT16SF2/MPPLPPT16SR2 (Supplementary Table S1, [30]) as well as Sl-ACT-F/Sl-ACT-R primers, respectively (Supplementary Table S1). To determine the fold changes in phytoplasma titer, the expression levels were normalized to the internal control. A minimum of three independent biological samples were used. The following thermocycling conditions for qPCR operation were used: an initial denaturation step at 95 °C for 3 min, followed by 40 cycles of amplification. Each cycle included denaturation at 95 °C for 5 s, annealing, and extension at 60 °C for 10 s. Statistical analysis was conducted using a Student’s *t* test in Microsoft Excel (Microsoft Corporation, Seattle, WA, USA) to assess the significance of differences between groups.

### 2.10. Gene Knockdown in Tomato Plants by Tobacco Rattle Virus (TRV) Vector

To knock down the tomato gene BiP, the gene fragments were amplified from tomato plant DNA using the specific primer pair TRV-Sl-BiP-F/TRV-Sl-BiP-R (Supplementary Table S1), respectively. The amplified gene fragment was then cloned into the pDONR221 entry vector (Thermo Fisher Scientific, Waltham, MA, USA) using BP Clonase II enzyme (Thermo Fisher Scientific, Waltham, MA, USA) to create a pDONR221-BiP plasmid clone. Next, the BiP fragment was cleaved from the pDONR221 vector using *Hpa*I and *EcoR*V restriction enzymes (NEB, Ipswich, MA, USA) and cloned into the vector YL279 using LR Clonase II enzyme (Thermo Fisher Scientific, Waltham, MA, USA). YL279 is a pTRV2 vector that is commonly used for virus-induced gene silencing in plants and was obtained from the Arabidopsis Biological Resource Center (ABRC, Columbus, OH, USA). The resulting plasmid constructs, pTRV2-BiP and pTRV2-GFP (as a control), were transformed into agrobacterium strain C58C1, which was given by Dr. Rose Hammond. Two-week-old tomato plants were then agroinfiltrated with the TRV constructs. Ten days after the TRV inoculation, the tomato plants were infected with PPT phytoplasma via graft inoculation. Newly grown leaves were harvested one month after the PPT phytoplasma infection, and both DNAs and RNAs were extracted for further analysis.

## 3. Results

### 3.1. PPT Phytoplasma Infection Induced Excessive Accumulation of ER-Resident Proteins and Caused ER Stress in Tomato Plants

**Figure 1:**
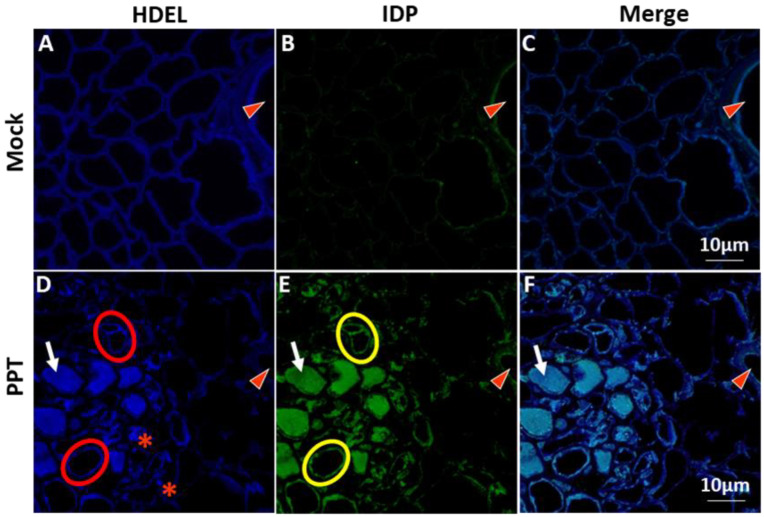
Confocal microscopy for visualization of the immunostained endoplasmic reticulum (ER) network and potato purple top (PPT) phytoplasmas in tomato plants. The figure provides critical visual evidence of the cellular interactions between the immunostained ER network and PPT phytoplasmas in tomato plants. (**A**–**C**) mock control plants; (**D**–**F**) PPT-phytoplasma-infected tomato plants. HDEL, as the marker of ER lumen, was used to visualize the ER network structure in mock control (**A**) and infected (**D**) plants. Immunostaining was performed using an anti-HDEL antibody and a secondary antibody conjugated with Alexa Fluor™ 488 (Blue). The distribution and colonization of PPT phytoplasmas were observed in mock control (**B**) and infected (**E**) plants. Immunostaining was conducted with an anti-immunodominant membrane protein (IDP) antibody and a secondary antibody conjugated with Alexa Fluor™ 405 (green). (**C**,**F**) are the merged images of (**A**) and (**B**), and (**D**) and (**E**), respectively. Red triangles indicate the xylem tissues. White arrows indicate aggregated protein structures. In some infected phloem cells (sieve element and/or companion cell, represented by red circles in (**D**)), the HDEL signals were detected on the edge of the cell, appearing to be anchored to the plasma membrane. IDP signals were also detected near the plasma membranes in the same cells represented by yellow circles in (**F**). In some instances, HDEL signals were found within the cytoplasm of the cell (indicated by the red asterisk). Scale bar = 10 µm.

HDEL signal serves as an indicator of ER homeostasis and can also be utilized to visualize the ER network structure ([24,25], for details, see Section 1, Introduction). In this study, the abundance and localization of HDEL-containing proteins in the ER lumen of plants infected with PPT phytoplasma were investigated. To accomplish this, paraffin sections of cross-stem samples from the infected plants were examined using confocal microscopy with an anti-HDEL primary antibody and a secondary antibody that produces a blue fluorescence signal. As shown in Figure 1, intense blue HDEL signals were observed in infected plants compared to mock plants (Figure 1A,D, Supplementary Table S2), indicating the excessive accumulation of HDEL-containing proteins in the ER lumen of infected plants. Moreover, a notable difference was observed in the ER network structure between infected and mock control plants. Instead of a well-organized and interconnected network of tubules and cisternae (Figure 1A) found in mock control plants, the number of tubules was significantly reduced in infected plants, and a disrupted/collapsed ER network appeared as highly dense structures (Figure 1D), likely formed by protein aggregates (marked with white arrows in Figure 1D).

In addition to the presence of protein-aggregated high-density structures, scattered HDEL signals were also detected in the infected phloem including sieve elements and companion cells. These signals were observed both around the edges, seemingly anchored to the plasma membrane (see the red circles in Figure 1D), and floating within the cytoplasm of the infected cells (marked with red asterisks in Figure 1D). This scattered distribution of HDEL signals indicates significant alterations in the distribution and localization of ER components within the infected cells. Taken together, these findings highlight the impact of phytoplasma infection on the organization and functionality of the ER. The collapse of the ER network, the presence of protein aggregates, and the scattered distribution of ER markers all reflect disruptions in ER homeostasis and the induction of ER stress. These alterations likely contribute to the overall disturbance of cellular processes within infected plants and have implications for phloem function and nutrient transport.

**Figure 2:**
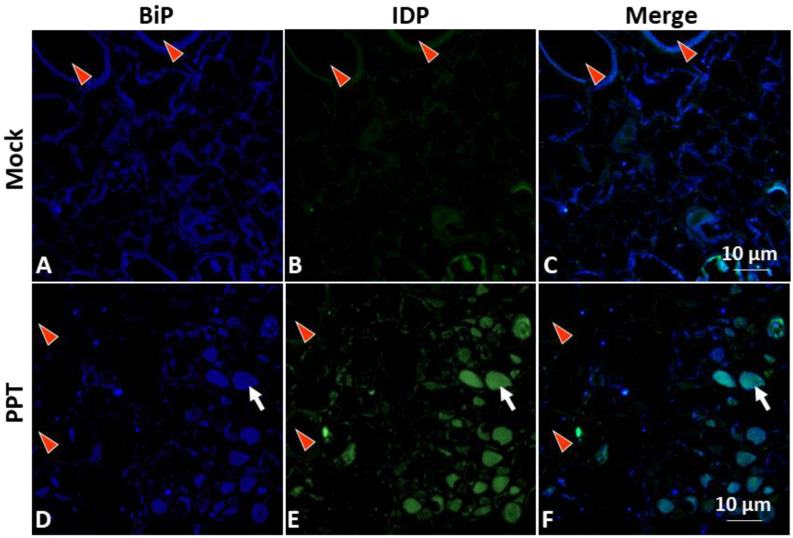
Confocal microscopy for visualization of the immunostained ER luminal binding immunoglobulin protein (BiP, an ER-resident protein) and potato purple top (PPT) phytoplasmas in tomato plants. The figure provides critical visual evidence of the cellular interactions between BiP and PPT phytoplasmas in tomato plants. (**A**–**C**) mock control plants; (**D**–**F**) PPT-phytoplasma-infected tomato plants. Immunostaining was performed using an anti-BiP antibody and a secondary antibody conjugated with Alexa Fluor™488 (Blue) to visualize the BiP protein expression in mock control (**A**) and PPT-phytoplasma-infected tomato plants (**D**). The distribution and colonization of PPT phytoplasmas were observed in mock control (**B**) and infected (**E**) plants. Immunostaining was conducted with an anti-immunodominant membrane protein (IDP) antibody and secondary antibody conjugated with Alexa Fluor™ 405 (green). (**C**,**F**) are the merged images of (**A**) and (**B**), and (**D**) and (**E**), respectively. Red triangles indicate the xylem tissues. White arrows indicate the large, aggregated structures. Scale bar = 10 µm.

ER luminal binding immunoglobulin protein (BiP), an ER-resident protein, was investigated to further understand the involvement of ER organelle and its resident proteins in phytoplasma infection. Confocal microscopy, along with an anti-BiP antibody, was utilized to examine the localization and abundance of BiP in infected plants (Figure 2, Supplementary Table S3). The results revealed that BiP signals in the infected phloem were concentrated/aggregated with slightly higher intensity (Figure 2D) in contrast to those in the control plants (Figure 2A, Supplementary Table S3). These differential BiP signal profiles closely resembled the HDEL signal profiles described above (Figure 1D, Supplementary Table S2). The observed differential BiP signal distribution profiles were consistent with the results obtained from a qRT-PCR-based gene expression assay and a Western blot analysis. Both assays demonstrated increased expression of BiP in infected plants (further details are provided later in the results section). Taken together, these data indicate that the elevated level of BiP in the stressed and disrupted ER was induced by the phytoplasma infection. The increased abundance of BiP may reflect the plant response to ER stress caused by the infection. It is important to note that BiP serves both as an ER-resident protein and an essential ER chaperone, playing a critical role in protein folding and quality control within the ER. Therefore, the upregulation of BiP at both transcript and protein levels in infected plants highlights its significance in managing the protein folding processes and maintaining (and/or restoring) ER homeostasis during phytoplasma infection.

**Figure 3:**
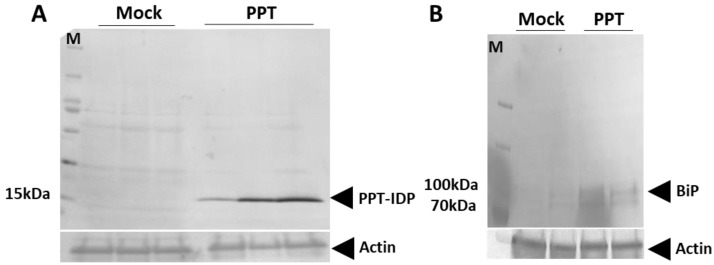
Western blot analysis results. Confirmation of the specificity of the antibody and investigation of the expression of the host protein in the tomato plants infected with potato purple top (PPT) phytoplasma. (**A**) Validation of the specificity of a polyclonal antibody raised against the purified immunodominant protein (IDP) of PPT phytoplasma. The total proteins were extracted from the leaves of tomato plants infected with PPT phytoplasma and mock control plants, and an anti-PPT-IDP antibody was used and confirmed its specificity in detecting IDP. (**B**) Determination of the expression of binding immunoglobulin protein (BiP) in PPT-phytoplasma-infected tomato plants. Actin antibody was used as the loading control. M: marker (PageRuler™ Plus Prestained Protein Ladder, 10 to 250 kDa).

In addition, to gain insights into the potential interactions of phytoplasma and the ER, the distribution and localization of phytoplasmas within host cells were examined. Specifically, the immunodominant protein of PPT phytoplasma (PPT-IDP) was expressed in *E. coli* and purified, and polyclonal antibodies against the PPT-IDP were raised in rabbits (Section 2, Materials and Methods). The specificity of the anti-PPT-IDP antibody was confirmed by Western blot analysis (Figure 3A). The serial paraffin sections used for the ER-resident protein study were stained with the anti-PPT-IDP antibody and a fluorescence-labeled secondary antibody, and the localization and distribution of PPT phytoplasma were examined using confocal microscopy. The results revealed that the PPT phytoplasma (green signals in Figure 1F and Figure 2F, Supplementary Tables S2 and S3) and a high abundance of ER-resident proteins (including BiP) (blue signals in Figure 1D and Figure 2D, Supplementary Tables S2 and S3) were colocalizing at the same subcellular compartment (Figure 1F and Figure 2F). It would be interesting to learn whether such colocalization suggests that PPT phytoplasma may have the ability to attract and potentially manipulate the ER organelle to support its own multiplication and/or survival within its host cells.

### 3.2. PPT Phytoplasma Induced Abnormal ER Morphology in Phloem

**Figure 4:**
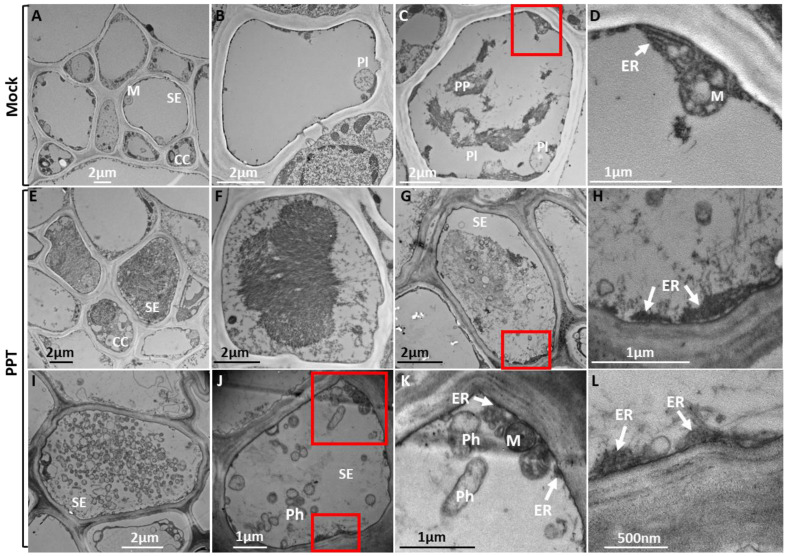
Transmission electron microscopy for visualization of morphological changes in the ultrastructure of endoplasmic reticulum (ER) organelle induced by potato purple top (PPT) phytoplasmas in tomato plants. (**A**–**D**) Mock control plants. (**D**) The close-up image of the red box of (**C**). (**E**–**L**) PPT-phytoplasma-infected tomato plants. (**H**) The close-up image of the red box of (**G**). (**K**,**L**) are close-up images of the upper and lower red boxes in (**J**). M: Mitochondria; PP: Phloem proteins; SE: sieve element; CC: companion cell; ER: endoplasmic reticulum; Ph: phytoplasma; Pl: plastid.

The ER morphological changes in the infected phloem were also analyzed by transmission electron microscopy (TEM) using the main stem sections. It is well known that the phloem is a specialized tissue that undergoes significant changes in its structure to minimize cellular infrastructure. Such structural changes result in mature sieve elements that lack a nucleus, vacuoles, ribosomes, and Golgi, making space for the translocation of photosynthates and other organic compounds. However, they still retain some organelles, such as the smooth ER, mitochondria, sieve element plastids, and phloem proteins (P proteins), which are essential for phloem function [31]. Consistent with the above description, typical rod-shaped mitochondria, P proteins, and plastids were observed in the sieve elements of mock control plants (Figure 4A–C). In contrast, in the phloem of infected plants, phytoplasma cells, abnormal ER, and electron-dense protein agglomerations were visualized (Figure 4E–L). Development of abnormally shaped ER (disrupted ER stack) was observed only in PPT-phytoplasma-infected sieve elements (Figure 4H,L), not in mock control plants (Figure 4D). Additionally, the highly dense protein-aggregated structures observed in the PPT-phytoplasma-infected sieve elements were likely formed from the accumulation of P proteins and misfolded proteins due to phytoplasma-infection-induced ER stress (Figure 4E–G). These protein-aggregated structures were complex and different from those seen in mock control plants (Figure 4C). Furthermore, in the infected phloem, some phytoplasmas were observed in the center of sieve elements, along with P proteins and electron-dense structures (Figure 4G). In other instances, a significant number of phytoplasma cells occupied the entire sieve element (Figure 4I), while some were scattered throughout the cell (Figure 4J). Interestingly, certain phytoplasma cells were found near the ER–mitochondria contact site, undergoing morphological changes resembling cell division or elongation (Figure 4J,K).

### 3.3. Activation of UPR in PPT-Phytoplasma-Infected Tomato Plants

**Figure 5:**
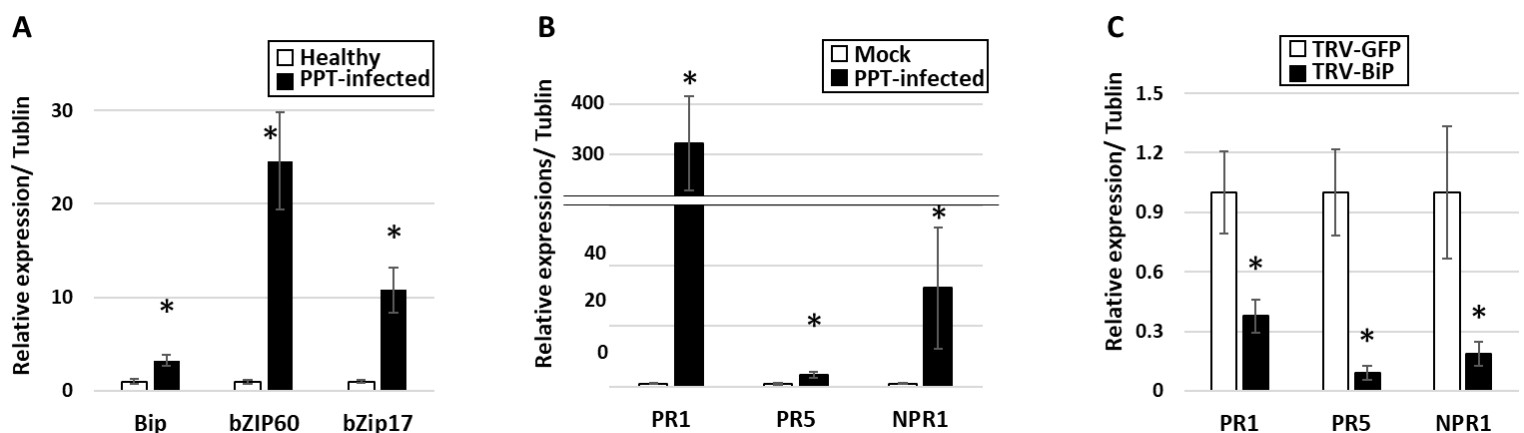
Gene expression analysis by quantitative real-time PCR (qRT-PCR) in tomato plants and BiP gene-silencing tomato line infected with potato purple top (PPT) phytoplasma. (**A**) Expression of UPR marker genes, BiP, bZIP60, and bZIP17, in PPT-infected tomato plants. (**B**) Expression of salicylic acid resistance marker genes, PR1, PR5, and NPR1, in PPT-phytoplasma-infected plants. (**C**): Expression of SA resistance marker genes, PR1, PR5, and NPR1, in PPT-phytoplasma-infected TRV-BiP tomato plants. Asterisk (*) indicates significantly different by Student’s *t* test (*p* < 0.05).

It is known that ER stress and consequent accumulation of misfolded and unfolded proteins can activate the UPR signaling pathway, which promotes protein folding, degradation, and transport to restore ER function [32]. To examine whether phytoplasma-infection-induced ER stress can trigger UPR activation, the expression levels of the UPR marker genes were assessed by qRT-PCR. These marker genes include BiP, basic leucine Zipper domain-containing transcription factor 60 (bZIP60), and bZIP17. Among them, BiPs are molecular chaperones located in the ER that play a key role in UPR. BiPs bind to unfolded or misfolded proteins and promote their refolding or targeted degradation through the ER-associated degradation (ERAD) pathway [33]. Increased expression of BiPs is a marker of ER stress and UPR activation. bZIP60 and bZIP17 are transcription factors that are activated in response to UPR signaling [34]. The transcriptional levels of the three UPR marker genes, BiP, bZIP60, and bZIP17, were significantly higher in plants infected with PPT phytoplasma than those in mock plants (Figure 5A). For instance, compared with mock controls, the expression level of BiP was found to be upregulated by approximately three folds in infected plants. This result was further confirmed through Western blot analysis and confocal microscopy utilizing an anti-BiP antibody, which revealed a significant increase in BiP protein expression in infected plants, providing additional support for the upregulation of BiP during phytoplasma infection (Figure 2D and Figure 3B). These findings provided evidence that during PPT phytoplasma infection, there is an induction of ER stress in plants, which in turn triggers UPR signals and up-regulates the expression of UPR target genes.

### 3.4. Do Phytoplasmas Utilize ER and Its Resident Proteins for Their Own Infection and Multiplication?

Intracellular pathogens, especially viruses, have been observed to target the ER of host cells to create a replication niche and influence the host immune response [35]. Phytoplasmas were also able to induce morphological changes in host ER ([20,21], and colocalize with the ER-resident proteins (this study)). However, it remains unclear whether the phytoplasma uses the ER and its resident protein for their survival and multiplication. To gain further understanding of this mechanism, this study sought to enhance and suppress UPR activity/ER-resident protein to manipulate ER organelle and investigate their effects on plant phytoplasma interactions, thereby establishing whether ER and UPR play crucial roles in phytoplasma multiplication and host resistance. In the current study, salicylic acid (SA)-dependent signaling was used as a marker of host resistance due to its demonstrated defensive role in PPT phytoplasma infection in our previous study [30] and this study (Figure 5B).

**Figure 6:**
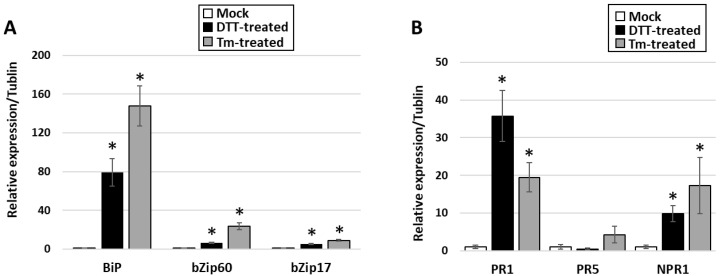
Gene expression analysis by quantitative real-time PCR (qRT-PCR) in potato purple top (PPT)-phytoplasma-infected plants treated with UPR chemical inducers (Tm and DTT). The figure provides information on the effects of UPR chemical inducers on the expression of specific genes related to the UPR and salicylic acid (SA) resistance pathways. (**A**) UPR marker genes, BiP, bZIP60, and bZIP17. (**B**) SA resistance marker genes, PR1, PR5, and NPR1. Asterisk (*) indicates significantly different by Student’s *t* test (*p* < 0.05).

**Figure 7:**
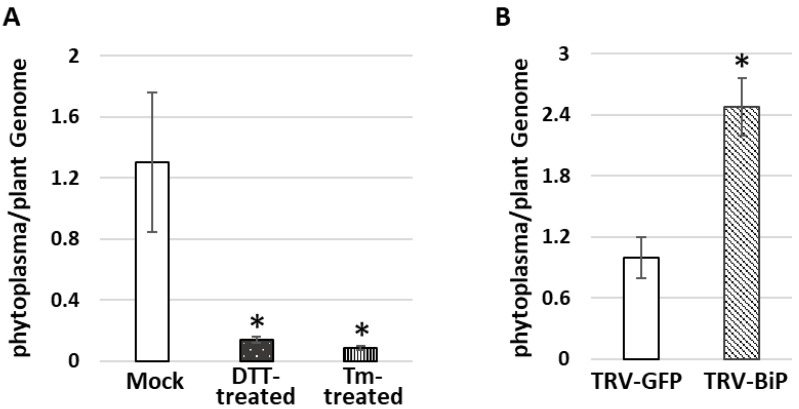
Measurement of phytoplasma titer in different tomato plants infected with potato purple top (PPT) phytoplasma. The figure provides important data on phytoplasma titer changes in different experimental conditions. (**A**) PPT phytoplasma titer in PPT-infected tomato plants treated with UPR chemical inducers DTT and Tm. (**B**) PPT phytoplasma titer in BiP gene-silencing tomato line (TRV-BiP), which was generated by using a tobacco rattle virus (TRV) vector. TRV-GFP tomato line was used as a control. Asterisk (*) indicates significantly different by Student’s *t* test (*p* < 0.05).

**Figure 8:**
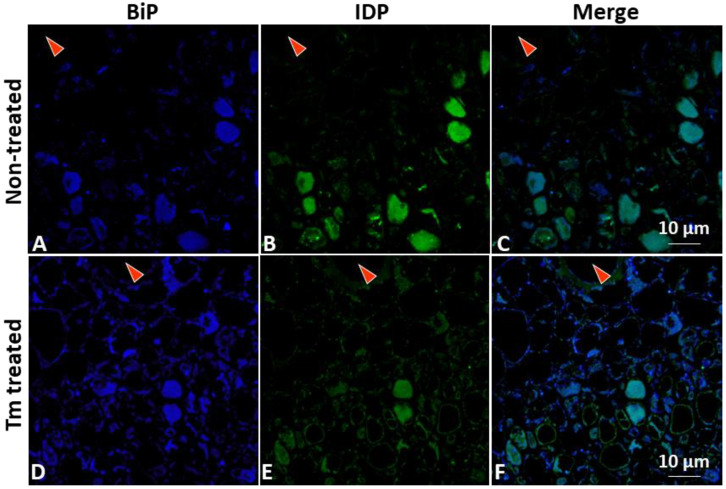
Confocal microscopy for visualization of the immunostained BiP and potato purple top (PPT) phytoplasmas in infected tomato plants treated with UPR chemical inducer. The figure provides a comprehensive view of the spatial relationship between BiP and PPT phytoplasmas within the infected tomato plants treated with UPR chemical inducer. (**A**–**C**) PPT-phytoplasma-infected tomato plants in ½ MS medium (without Tm treatment, serving as control plants for UPR chemical inducer Tm induction experiment). (**D**–**F**) Tm-treated tomato plants infected with PPT phytoplasma. BiP: ER luminal binding protein, chaperone, and ER-resident protein. Immunostaining was performed using an anti-BiP antibody and a secondary antibody conjugated with Alexa Fluor™ 488 (Blue). BiP protein expression was observed in Tm-untreated infected plants (**A**) and Tm-treated infected plants (**D**). The distribution and colonization of PPT phytoplasmas were observed in Tm-untreated infected plants (**B**) and Tm-treated infected plants (**E**). Immunostaining was conducted with an anti-immunodominant membrane protein (IDP) antibody and secondary antibody conjugated with Alexa Fluor™ 405 (green). (**C**,**F**) are the merged images of their left two images, respectively. Red triangles indicate the xylem tissues. Scale bar = 10 µm.

UPR was artificially activated by chemical inducers of the UPR such as dithiothreitol (DTT) and tunicamycin (Tm). Five days following treatment with DTT and Tm, the PPT-phytoplasma-infected plants displayed necrosis, indicating that these chemical inducers were effective. Moreover, qRT-PCR results revealed that UPR marker genes (BiP, bZIP60, and bZIP17) and downstream SA-dependent resistance marker genes (NPR1, PR1, and PR5) were significantly increased within one day of treatment with the chemical inducers (Figure 6A,B). Importantly, the accumulation levels of PPT phytoplasma in the treated infected leaves were markedly lower than those in the nontreated control samples (Figure 7A). Furthermore, ER-resident proteins in the PPT-infected plants treated with UPR chemical inducer were examined using confocal microscopy. Compared to the nontreated controls, the Tm-treated infected plants showed a higher abundance of HDEL signals using an anti-HDEL antibody (Supplementary Figure S1 and Table S5). In addition to HDEL retention proteins, based on immunostaining with an anti-BiP antibody and a fluorescence-labeled secondary antibody, the confocal microscopic studies also showed that the BiP expression increased in Tm-treated infected plants in contrast to nontreated controls (Figure 8). Enhancement of BiP expression in PPT-phytoplasma-infected plants at both transcript and protein levels have been confirmed in this study by qRT-PCR and Western blot analysis (Figure 3B and Figure 5A, Supplementary Table S3). Consistent with our qPCR results of reduced phytoplasma titer in infected plants treated with UPR inducers (Figure 7A), confocal microscopy studies showed a substantially lower population of phytoplasma colonization in infected plants treated with Tm (Figure 8E and Figure S1E, Tables S4 and S5).

In addition, the BiP gene in tomato plants was knocked down by virus-induced gene silencing (VIGS) technique with a tobacco rattle virus (TRV) vector. The suppression of BiP expression was confirmed by qRT-PCR. In the TRV-BiP gene-silencing tomato line infected with PPT phytoplasma, the phytoplasma titer was 2.5-fold higher than in TRV-GFP control plants (Figure 7B). Additionally, SA-dependent defense pathway marker genes, including NPR1, PR1, and PR5, were decreased (Figure 5C). These results suggest that the suppression of BiP expression compromised the plant’s ability to mount an effective defense against PPT phytoplasma infection.

In summary, two parallel lines of data provide evidence that there is a relationship between ER stress/UPR activity, phytoplasma colonization, and SA-dependent defense in plants. On one hand, ER stress and increased UPR activity have been shown to enhance SA-dependent defense and inhibit phytoplasma proliferation. On the other hand, suppression of the BiP gene leads to decreased SA-dependent defense and increased phytoplasma titer in plants.

## 4. Discussion

The ER plays a significant role in the replication and survival of various intracellular pathogens, including bacteria and viruses. These pathogens have evolved sophisticated strategies to exploit the ER and its associated processes to establish and maintain infection within host cells. Bacterial pathogens often manipulate the ER to create a favorable niche for their replication. For example, *Candidatus* Liberibacter asiaticus (CLas) is a phloem-restricted bacterium responsible for citrus greening disease, and it is transmitted by *Diaphorina citri* (Asian citrus psyllid). Recent research has revealed that CLas directs these structures to specific compartments known as Liberibacter-containing vacuoles, where the bacterium undergoes reproduction within the host (insect vector) cells [36]. Additionally, *Salmonella enterica* serovar Typhimurium remodels the ER membrane to form a modified phagosome known as Salmonella-containing vacuole (SCV), which provides a protected environment for bacterial replication and evasion of host immune responses [37]. Similarly, *Legionella pneumophila*, the causative agent of Legionnaires’ disease can utilize the ER to create a specialized compartment called the Legionella-containing vacuole (LCV). The LCV serves as a site for bacterial replication and the acquisition of essential nutrients from the host [38].

Intracellular bacteria can also exploit ER-associated processes to their advantage. For instance, Chlamydia species, including *Chlamydia trachomatis*, utilize the ER to establish a replication niche known as the inclusion, where they replicate and survive within a modified vacuole. Chlamydia actively manipulates the ER to support its own intracellular survival and subverts the UPR of host cells to maintain ER homeostasis [39,40]. Viruses also rely on the ER for various stages of their life cycle. Many enveloped viruses including the flaviviruses utilize the ER for viral protein synthesis, folding, and assembly. ER-derived membranes, such as the endoplasmic reticulum–Golgi intermediate compartment (ER–GIC) and the ER–Golgi interface, serve as platforms for viral replication and the assembly of viral particles [41]. In addition, certain plant viruses such as turnip mosaic virus (TuMV) and TMV induce the formation of vesicles or membranous structures derived from the ER to serve as sites for viral RNA replication. These replication complexes provide a protected environment where viral RNA can be synthesized and replicated efficiently [16,42].

Phytoplasmas, as intracellular plant pathogens, also highly depend on host cells for their survival and propagation because they cannot synthesize many essential molecules [6,7]. The ER, with its crucial roles in protein synthesis, folding, and trafficking, presents an attractive target for phytoplasmas to exploit. In this study, the impact of PPT phytoplasma infection on the ER of tomato plants was investigated using both morphological and molecular analyses. These analyses aimed to elucidate the relationships between phytoplasmas and the ER, providing insights into how phytoplasmas may also exploit the ER for their own benefit. TEM and confocal microscopy-based morphological analysis revealed the ER structural changes and altered localization of ER-resident proteins in response to PPT phytoplasma infection (Figure 1D, Figure 2D and Figure 4H,L). These alterations suggest a potential remodeling of the ER, which involves the activation of signaling pathways such as the UPR (Figure 5). The remodeling of the ER likely plays a crucial role in facilitating the establishment and persistence of phytoplasma infections within host plants. Beyond the evident morphological changes and protein localization alterations, the reprogramming of the ER during phytoplasma infection likely encompasses broader aspects. It may involve changes in ER membrane dynamics, modifications in lipid composition, and alterations in ER-associated processes such as calcium signaling and stress response pathways. These complex alterations in the ER could be envisioned as phytoplasma’s adaptation to the intracellular environment and establishment of a favorable niche for their survival, multiplication, and evasion of host defense mechanisms.

The association between phytoplasmas and the ER–mitochondria contact site, along with the observed morphological changes such as elongation and division of phytoplasma cells near this region (Figure 4J,K), suggests their potential interactions with both the ER and mitochondria. Elongation and division are well-known characteristics of bacteria reproduction [43]. These observations indicate the involvement of the host ER and mitochondria in phytoplasma cell division or morphological changes necessary for organelle attachment and further interactions.

Our study also revealed the presence of highly dense protein aggregates in infected plants, as visualized through both TEM and confocal microscopy analyses (Figure 1D–F, Figure 2D–F and Figure 4E,F). These aggregates, which may consist of P-proteins, misfolded or unfolded proteins resulting from phytoplasma infection, and other cellular components, represent an interesting aspect of phytoplasma–host interactions. The formation of these highly dense protein aggregates within infected cells raises questions regarding their role and significance. One possibility is that these aggregates serve as a defense mechanism deployed by the host plant to sequester and immobilize the pathogen or its components. By localizing the pathogen within these aggregates, the host plant may effectively limit the spread and impact of the infection. Another intriguing hypothesis is that these protein aggregates may serve as sites for the accumulation or aggregation of phytoplasma proteins or other cellular components. Such aggregation could potentially play a role in facilitating phytoplasma multiplication, protecting pathogen components from host defenses, or serving as a reservoir for phytoplasma assembly and persistence within the host.

The presence of these highly dense protein aggregates highlights the complex interplay between phytoplasmas and their host plants. Elucidation of the molecular mechanisms underlying the formation, composition, and functional significance of these aggregates will provide valuable insights into the strategies employed by phytoplasmas to colonize and manipulate their host plants. Further investigations are warranted to explore the specific proteins and factors involved in the formation of these aggregates and to interpret their impact on phytoplasma pathogenicity.

In addition to morphological analysis, a comprehensive molecular analysis was conducted to explore the impact of ER organelle and its resident protein on phytoplasma multiplication and survival. Specifically, the focus was on examining the activation of the UPR signaling pathway, as well as downstream SA signaling pathways to understand their relationship with phytoplasma colonization. In UPR chemical-inducer-treated PPT-phytoplasma-infected tomato plants, the expression of UPR marker genes (BiP, bZIP60, and bZIP17) and downstream SA-dependent resistance marker genes (NPR1, PR1, and PR5) was significantly increased (Figure 6). Importantly, the accumulation levels of PPT phytoplasma in treated infected leaves were markedly lower compared to nontreated controls, demonstrating the suppressive effect of UPR induction on phytoplasma colonization (Figure 6, Figure 7A and Figure S1, Tables S4 and S5). Confocal microscopy analysis further supported these findings, showing a lower population of phytoplasma colonization in plants treated with UPR inducers (Figure 8 and Figure S1). Additionally, the treated infected plants exhibited a higher abundance of ER-resident proteins, as indicated by HDEL retention and increased expression of BiP (Figure 8 and Figure S1), suggesting the involvement of the ER in UPR activation and defense responses against phytoplasmas. Conversely, when the ER-resident protein BiP was silenced, the tomato line infected with PPT phytoplasma showed suppressed expression of SA marker genes (Figure 5C). These changes in gene expression were accompanied by an enhancement in phytoplasma titer (Figure 7), indicating that the suppression of BiP compromised the effective plant defense against phytoplasma infection.

In summary, the findings of this study unveil phytoplasma’s ability to exploit the ER and its resident proteins, allowing them to evade host defenses and utilize host cellular processes to create a favorable environment for their survival and multiplication. Understanding the molecular mechanisms that underlie such a phenomenon is crucial for devising novel strategies to combat phytoplasma diseases.

## 5. Conclusions

This study explores the interactions between the intracellular pathogen PPT phy-toplasma and the ER of its host plant cells. The PPT phytoplasma exploits the ER and its resident proteins for survival, leading to disrupted ER structures, abnormal protein ac-cumulation, and aggregate formation in the phloem. The colocalization of phytoplasma cells with ER-resident proteins suggests a direct linkage between phytoplasma infection and ER stress along with activation of UPR. The observed negative correlation between the UPR activation and phytoplasma titer indicates that UPR plays a role in restraining phytoplasma proliferation. The findings from this study provide insights into devising new strategies for managing phytoplasma-related diseases by targeting the ER-mediated host–pathogen dynamics.

## Supplementary Materials

The following supporting information can be downloaded at: https://www.mdpi.com/article/10.3390/cells12162110/s1, Figure S1: Confocal microscopy for visualization of the immunostained ER resident proteins and potato purple top (PPT) phytoplasmas in infected tomato plants treated with UPR chemical inducer; Table S1: Primers used in this study for antibody synthesis, phytoplasma titer measurement, gene expression assessment, and gene silencing construct; Table S2: Fluorescence intensity measurement of HDEL and IDP signals in PPT phytoplasma-infected and control plants based on histogram function of Adobe Photoshop software; Table S3: Fluorescence intensity measurement of Bip and IDP signals in PPT phytoplasma-infected and control plants based on histogram function of Adobe Photoshop software; Table S4: Fluorescence intensity measurement of Bip and IDP signals in PPT phytoplasma-infected tomato plants treated with Tunicamycin and non-treated controls based on histogram function of Adobe Photoshop software; Table S5: Fluorescence intensity measurement of HDEL and IDP signals in PPT phytoplasma-infected tomato plants treated with Tunicamycin and non-treated controls based on histogram function of Adobe Photoshop software.
