# Supplementary material for: The Endoplasmic Reticulum Is a Key Battleground between Phytoplasma Aggression and Host Plant Defense
Source: Cells. 2023 Aug 21;12(16):2110. doi: 10.3390/cells12162110 (PMC10453741; doi:10.3390/cells12162110)
Supplement: Supplementary file 1 [file cells-12-02110-s001.zip › cells-2545403-supplementary.pdf]

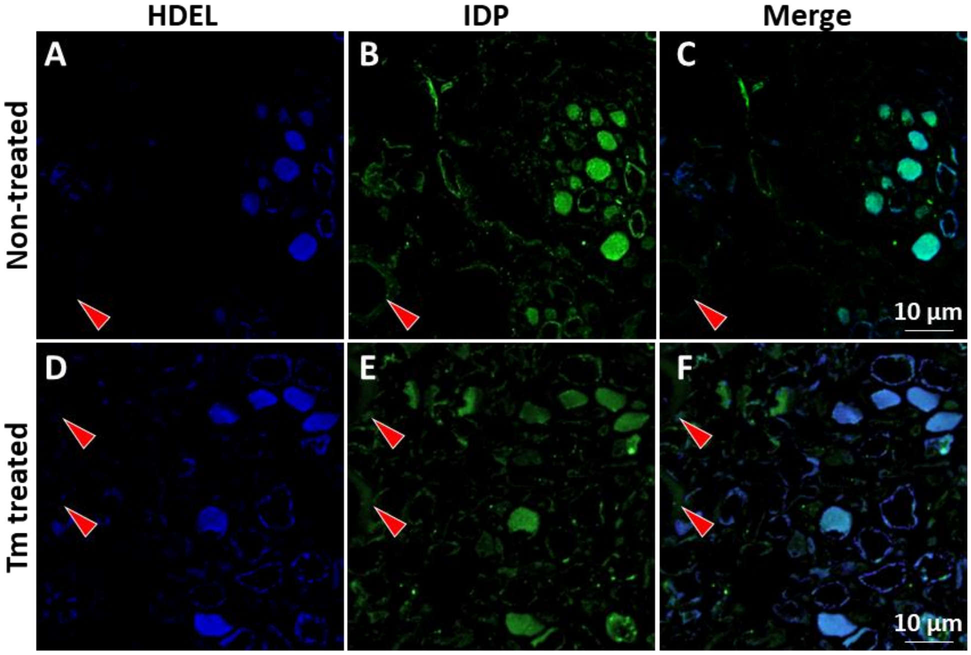

Supplementary Figure S1. Confocal microscopy for visualization of the immunostained ER resident proteins and potato purple top (PPT) phytoplasmas in infected tomato plants treated with UPR chemical inducer. (**A-C**): PPT phytoplasma-infected tomato plants in ½ MS medium (without Tm treatment, serving as control plants for UPR chemical inducer Tm induction experiment). (**D-F**): Tm-treated tomato plants infected PPT phytoplasma. (**A**) and (**D**): Immunostaining was performed using an anti-HDEL antibody and a secondary antibody conjugated with Alexa488 (Blue). The distribution and colonization of PPT phytoplasmas were observed Tm-untreated infected plants and Tm-treated infected plants; Immunostaining was conducted with anti-immunodominant membrane protein (IDP) antibody and secondary antibody conjugated with Alexa405 (green). (**C**) and (**F**) are the merged images of their left two images, respectively. Red triangles indicate the xylem tissues. Scale bar=10μm.

Supplementary Table S1. Primers used in this study for antibody synthesis, phytoplasma titer measurement, gene expression assessment, and gene silencing construct.

		PPT phytoplasma gene		Primer name	Primer sequence
Potato purple top (PPT) phytoplasma		Antibody synthesis for visualization of PPT phytoplasma	Gene encodes Immunodominant membrane protein (IDP)	PPT-IDP-F	GGGGACAAGTTTGTACAAAAAAGCAGGCT TCATGTCAAAGATTAAAGATTTTGTACAATC
				PPT-IDP-R	GGGGACCACTTTGTACAAGAAAGCTGGGT CTTATTTTTTTACAGACTCAGGTAATTTTTC
		Measurement of PPT phytoplasma titer by real-time PCR	16S ribosomal RNA (Wu et al., 2012)	MPPLPPT16SF2	AGGGTGCGTAGGCTGTTAGA
				MPPLPPT16SR2	TGCCTCAGCGTCAGTAAAGA
Tomato genes		Tomato ortholog of arabidopsis gene (GenBank accession number)		Primer name	Primer sequence
	Gene expression assessment by qRT-PCR	Pathogen related gene 1		Sl-PR1-F	AACTCGGTACGTCTTGGTTG
		(Sl-PR1, NM_001247429)		Sl-PR1-R	GACGTTGTCCTCTCCAGTTAC
		Pathogen related gene 5		Sl-PR5-F	TGTTAGCCTTGTCGATGGATAC
		(Sl-PR5, XM_004238172)		Sl-PR5-R	TCCTTTGGACAGATCGCATTTA
		NONEXPRESSOR OF PATHOGENESIS-RELATED GENES 1 (NPR1)		Sl-NPR1-F	ACGAAGCTGGAACTCAAAGAG
		(Sl-NPR1, NM_001247629)		Sl-NPR1-R	ACAAATAGGCGAGCACACTG
		Glucose-regulated protein 78 (BiP/grp78)		Sl-BiP-F	TGGAAAGGACATCAGCAAGG
		(Sl-BiP, NM_001247707)		Sl-BiP-R	TCAATCTCAACCCGGACTTG
		bZIP transcription factor 60 (bZIP60)		Sl-bZIP60-F	TTCCCTGCTTTGGTTCCTG
		(Sl-bZIP60, XM_004238421)		Sl-bZIP60-R	TGGTTTCTGCTTCCCTGAC
		bZIP transcription factor 17(bZIP17)		Sl-bZIP17-F	TGAGATATGGAGGTACGAGGG
		(Sl-bZIP17, XM_004249071)		Sl-bZIP17-R	CATCCACAACCAAAACTCTTCC
		Tubulin		Sl-TUB-F	AGCTCATTAGCGGCAAAGAA
		(Sl-TUB, XM_004232178)		Sl-TUB-R	AGTACCCCCACCAACAGCA
		Actin		Sl-ACT-F	TGTCCCTATTTACGAGGGTTATGC
		(Sl-ACT, NM_001321306)		Sl-ACT-R	CAGTTAAATCACGACCAGCAAGAT
	TRV construct	Glucose-regulated protein 78 (BiP/grp78) (Sl-BiP, NM_001247707)		TRV-Sl-BiP-F	GGGGACAAGTTTGTACAAAAAAGCAGGCTT CAGCTAATGACCAAGGAAACAGGATTACAC
				TRV-Sl-BiP-R	GGGGACCACTTTGTACAAGAAAGCTGGGT CTTCATCTTTGTCAAAATCATGGCGCTGA

Supplementary Table S2. Fluorescence intensity measurement of HDEL and IDP signals in PPT phytoplasma-infected and control plants based on histogram function of Adobe Photoshop software.

			Randomly selected 3 stained spots			3 spots (Mean)	Entire (Mean)
			spot 1	spot 2	spot 3		
HDEL (blue signals)	Mock control	Histogram reading	16.95	13.69	16.2	15.61±1.53	13.62
		Pixel	191	156	97	148±42.49	14302/29929
	PPT infected	Histogram reading	18.46	16.21	17.2	17.29±1.01	13.08
		Pixel	295	415	187	299±102.01	5612/29929
IDP (green signals)	Mock control	Histogram reading	ND	ND	ND	ND	ND
		Pixel	ND	ND	ND	ND	ND
	PPT infected	Histogram reading	68.47	69.58	64.65	67.57±2.31	41.53
		Pixel	344	187	412	314.33±103.21	6711/29929

Supplementary Table S3. Fluorescence intensity measurement of Bip and IDP signals in PPT phytoplasma-infected and control plants based on histogram function of Adobe Photoshop software.

			Randomly selected 3 stained spots			3 spots	Entire
			spot 1	spot 2	spot 3	(Mean)	(Mean)
Bip (blue signals)	Mock control	Histogram reading	12.17	13.34	11.37	12.29±0.89	10.99
		Pixel	119	102	105	108.67±8.12	6426/30276
	PPT infected	Histogram reading	14.13	14.65	13.49	14.09±0.52	11.56
		Pixel	190	148	142	160.00±23.39	3666/30276
IDP (green signals)	Mock control	Histogram reading	ND	ND	ND	ND	ND
		Pixel	ND	ND	ND	ND	ND
	PPT infected	Histogram reading	72.54	82.44	57.49	70.82±11.24	44.09
		Pixel	191	137	151	159.67±25.07	3690/30276

Supplementary Table S4. Fluorescence intensity measurement of Bip and IDP signals in PPT phytoplasma-infected tomato plants treated with Tunicamycin and non-treated controls based on histogram function of Adobe Photoshop software.

			Randomly selected 3 stained spots			3 spots (Mean)	Entire (Mean)
			spot 1	spot 2	spot 3		
Bip (blue signals)	Non-treated	Histogram reading	17.16	16.78	16.41	16.78±0.34	9.86
		Pixel	241	165	177	194.33±36.54	4996/30102
	Tm-Treated	Histogram reading	22.53	22.55	22.65	22.58±0.06	14.83
		Pixel	169	161	156	162.00±5.87	7635/30102
IDP (green signals)	Non-treated	Histogram reading	88.98	90.2	74.89	84.69±7.61	64.45
		Pixel	210	148	210	189.33±32.02	1915/30102
	Tm-Treated	Histogram reading	44.48	45.84	32.19	40.84±6.73	29.78
		Pixel	190	150	71	137.00±54.16	1903/30102

Supplementary Table S5. Fluorescence intensity measurement of HDEL and IDP signals in PPT phytoplasma-infected tomato plants treated with Tunicamycin and non-treated controls based on histogram function of Adobe Photoshop software.

			Randomly selected 3 stained spots			3 spots (Mean)	Entire (Mean)
			spot 1	spot 2	spot 3		
HDEL (blue signals)	Non-treated	Histogram reading	16.89	15.95	13.69	15.51±1.47	9.54
		Pixel	192	99	156	149.00±41.94	1539/29929
	Tm-Treated	Histogram reading	20.28	17.17	16.11	17.85±1.94	11.62
		Pixel	206	174	157	179.00±22.25	2717/29929
DP (green signals)	Non-treated	Histogram reading	106.77	101.45	81.64	96.62±11.84	96.06
		Pixel	184	142	113	146.33±31.93	732/29929
	Tm-Treated	Histogram reading	74.68	46.13	59	59.94±12.79	59.88
		Pixel	216	156	168	180.00±28.40	1949/29929
